# Thymic carcinoma with extrinsic occlusion of the left anterior descending artery: a distinctive case of myocardial infarction in a young woman

**DOI:** 10.1186/s43044-021-00178-1

**Published:** 2021-07-02

**Authors:** Ahmed Ayuna, Saad Ahmad, Sjirjel Alam, Nik Abidin

**Affiliations:** 1 Salford Royal NHS Foundation Trust, Manchester, UK; 2 Manchester Universities Foundation Trust, Manchester, UK

**Keywords:** Thymoma, Thymic carcinoma, TET, Coronary artery displacement, Myocardial ischemia, CT-FFR

## Abstract

**Background:**

Thymic epithelial tumour (TET) is the most common tumour affecting the anterior mediastinum in adults. The cardiac extension is often limited to the pericardium, and intracardiac extension is rare. We present a unique case of encasement and displacement of the left anterior descending coronary artery by the large mediastinal tumour leading to myocardial ischemia.

**Case presentation:**

Our patient is a 28-year-old lady with stage 4 TET. She presented with acute chest pain associated with 12-lead ECG changes and a significant rise in serial troponin I. Multimodality cardiac imaging revealed encasement and displacement of the left anterior descending coronary artery by the large mediastinal tumour. CT-FFR demonstrates evidence of ischemia which would account for her acute presentation. Following detailed MDT discussions between cardiologists, oncologists and cardiothoracic surgeons, the decision was made to treat this lady with palliative chemotherapy. Given the extent of the tumour invasion and failure of the initial therapy, her prognosis and the outcome were poor.

**Conclusions:**

TET could cause atrial compression, myocardial infiltration, and invasion of the pulmonary and caval veins; however, to the best of our knowledge, this is the first case reported of coronary artery displacement and encasement by TET.

**Supplementary Information:**

The online version contains supplementary material available at 10.1186/s43044-021-00178-1.

## Background

Thymic epithelial tumour (TET) is the most common tumour affecting the anterior mediastinum in adults [1]. However, they are still relatively rare tumours attributing from 0.2 to 1.5% of all malignancies [2]. TET most commonly affects adults who are between 40 and 60 years old, and the incidence is similar between males and females [3]. The cardiac extension is often limited to the pericardium, and intracardiac extension is rare [4]. Moreover, TET could be associated with distant metastasis to the liver, skin and lung (0-10%, mean 5.5%) [5]. In general, cardiac involvement in cancer patients can cause atrial compression, myocardial infiltration and invasion of the pulmonary and caval veins [6]. In thymic cancer, it has been reported to involve the right and left atrium, left ventricle and pulmonary veins [4]; however, to the best of our knowledge, this is the first case reported of coronary artery displacement and encasement by TET.

Nevertheless, coronary artery compression by a metastatic cardiac tumour resulting in acute coronary syndrome has been reported previously, and it was associated with poor outcome [7]. Thomas and colleagues have reported a case series of five cases of malignant thymoma with intracardiac extensions [4]. The cardiac extension involved the left ventricular outflow and caused haemodynamic compromise. Guidance for the definitive management of thymoma and thymic carcinomas is still premature due to the lack of randomised trials [8, 9]. Unfortunately, the majority of the patients with thymic carcinomas present with advanced-stage tumour [4]. The prognosis is mainly influenced by the stage and the complete resectability of the tumour, i.e. the higher the stage, the worse the prognosis [10]. In our case report, the patient was consented and all the data used was anonymised.

## Case presentation

A 28-year-old lady with metastatic thymic epithelial tumour (TET) presented to our hospital with worsening anginal chest pain. She was post-partum, having delivered a healthy baby girl just 7 weeks earlier. She has known malignancy with stage 4 thymic cancer, which has spread to the pleura and lymph nodes at the time of diagnosis and was managed with chemotherapy. Her initial diagnosis was made 4 months ago and confirmed with the tissue sample histopathology, which revealed an epithelial cancer of the thymus. The initial diagnosis was made when she was 6 months pregnant and developed rapidly progressive shortness of breath.

**Fig. 1:**
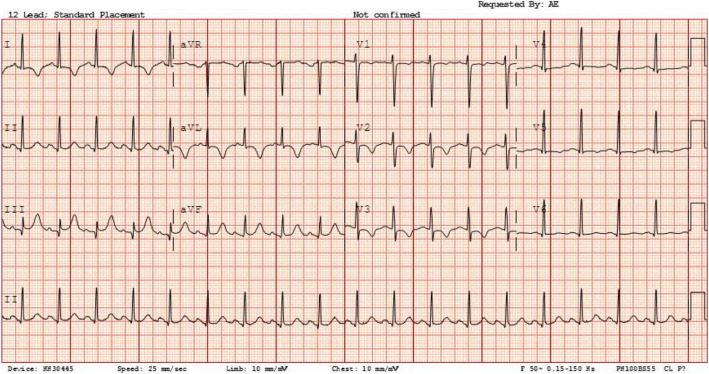
T-wave inversion of the anterior and lateral leads with saddle shape ST-elevation inferior leads with PR segment depression. The rhythm is sinus rhythm

Although her presentation this time was non-pleuritic chest pain, given her pre-existing co-morbidities, the main differential upon first assessment was that of pulmonary embolism. She had a raised D-dimer, and the fact that she has malignancy can increase the risk of thromboembolism. ECG, however, appeared ischaemic with ST depression and T-wave inversion across V1-V3 and in leads I and aVL (Fig. 1). Given this lady’s young age and lack of cardiovascular risk factors, acute coronary syndrome (ACS) was thought to be unlikely.

**Fig. 2:**
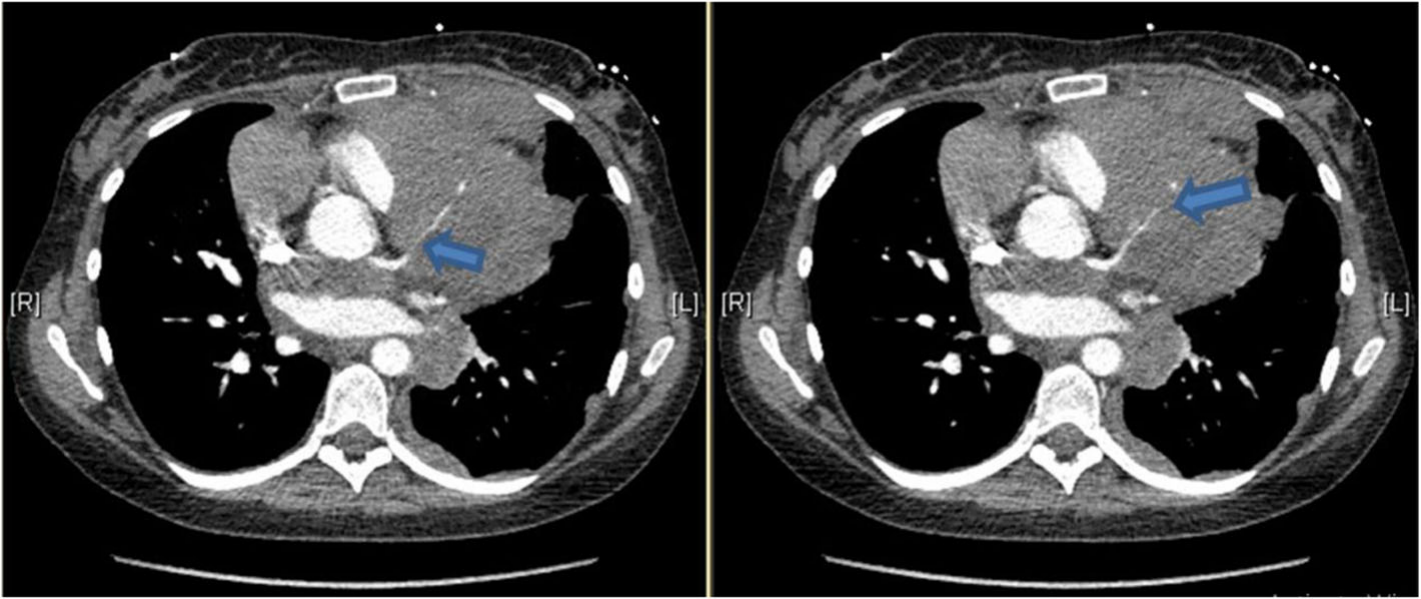
CT pulmonary angiogram reveals encasement and displacement of the left anterior descending coronary artery (blue arrows)

**Fig. 3:**
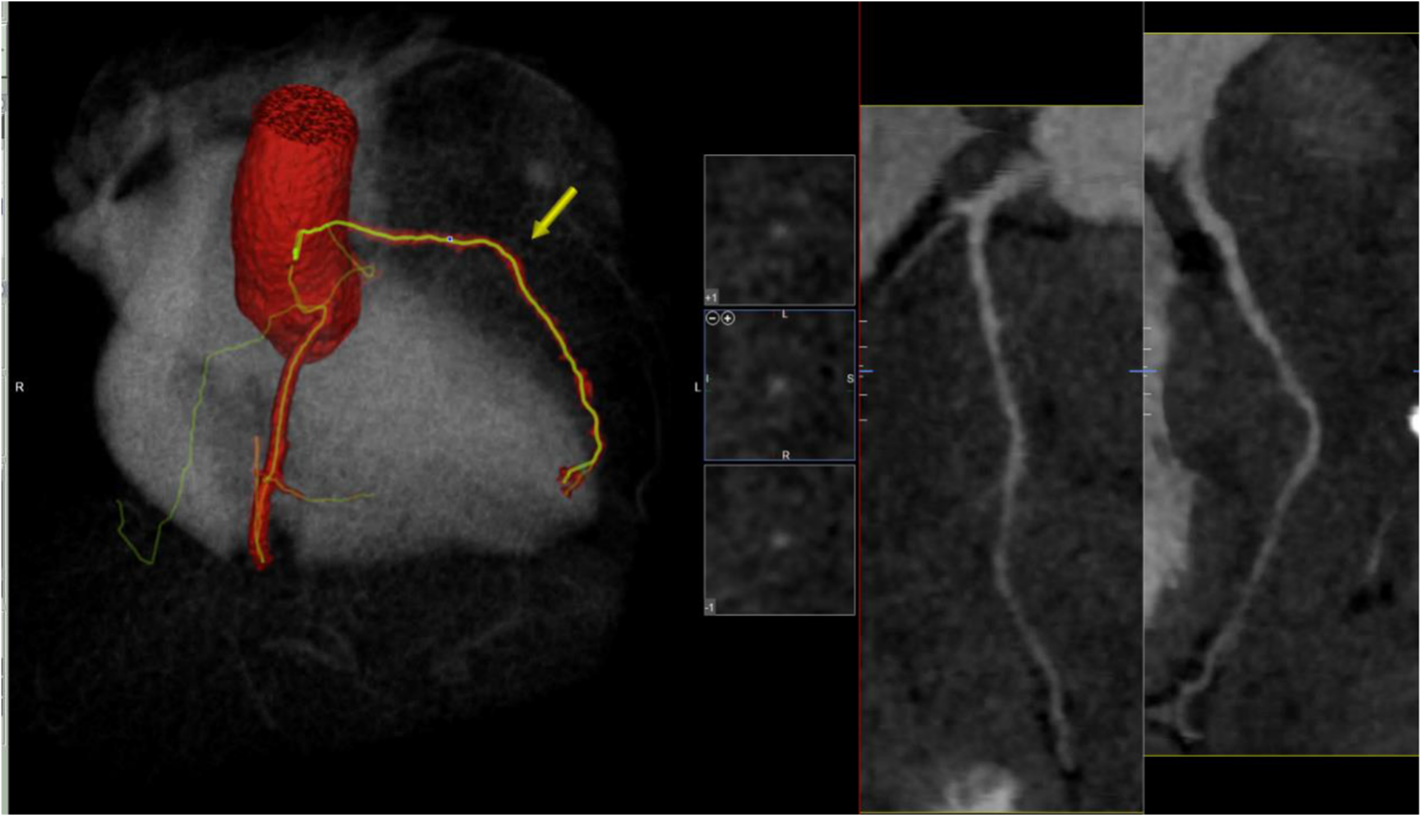
CT coronary angiogram reveals displacement of the left anterior descending coronary artery (LAD) yellow arrow. Right anterior oblique-cranial projection

**Fig. 4:**
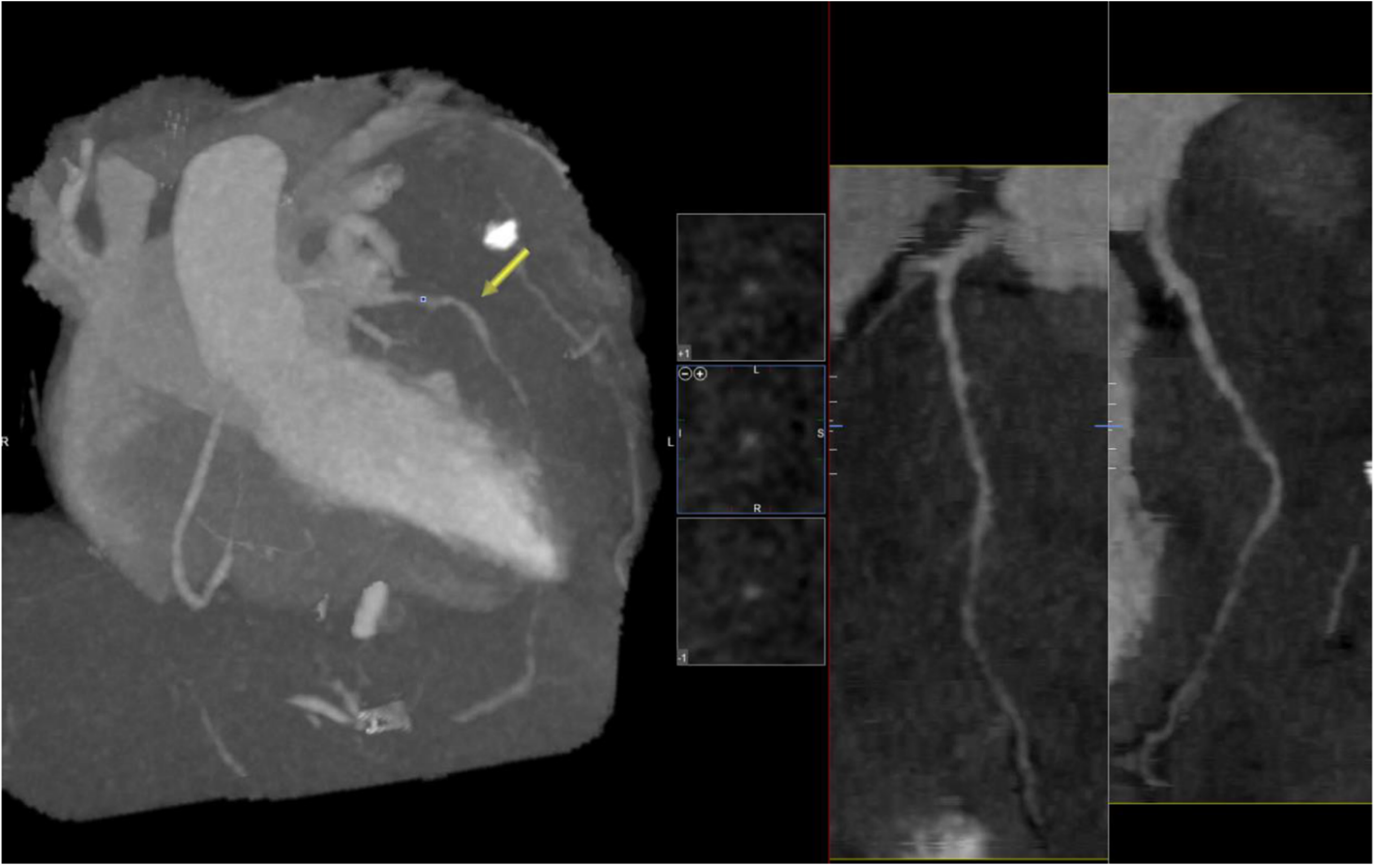
CT coronary angiogram reveals displacement of the left anterior descending coronary artery (LAD) yellow arrow. Right anterior oblique projection

**Fig. 5:**
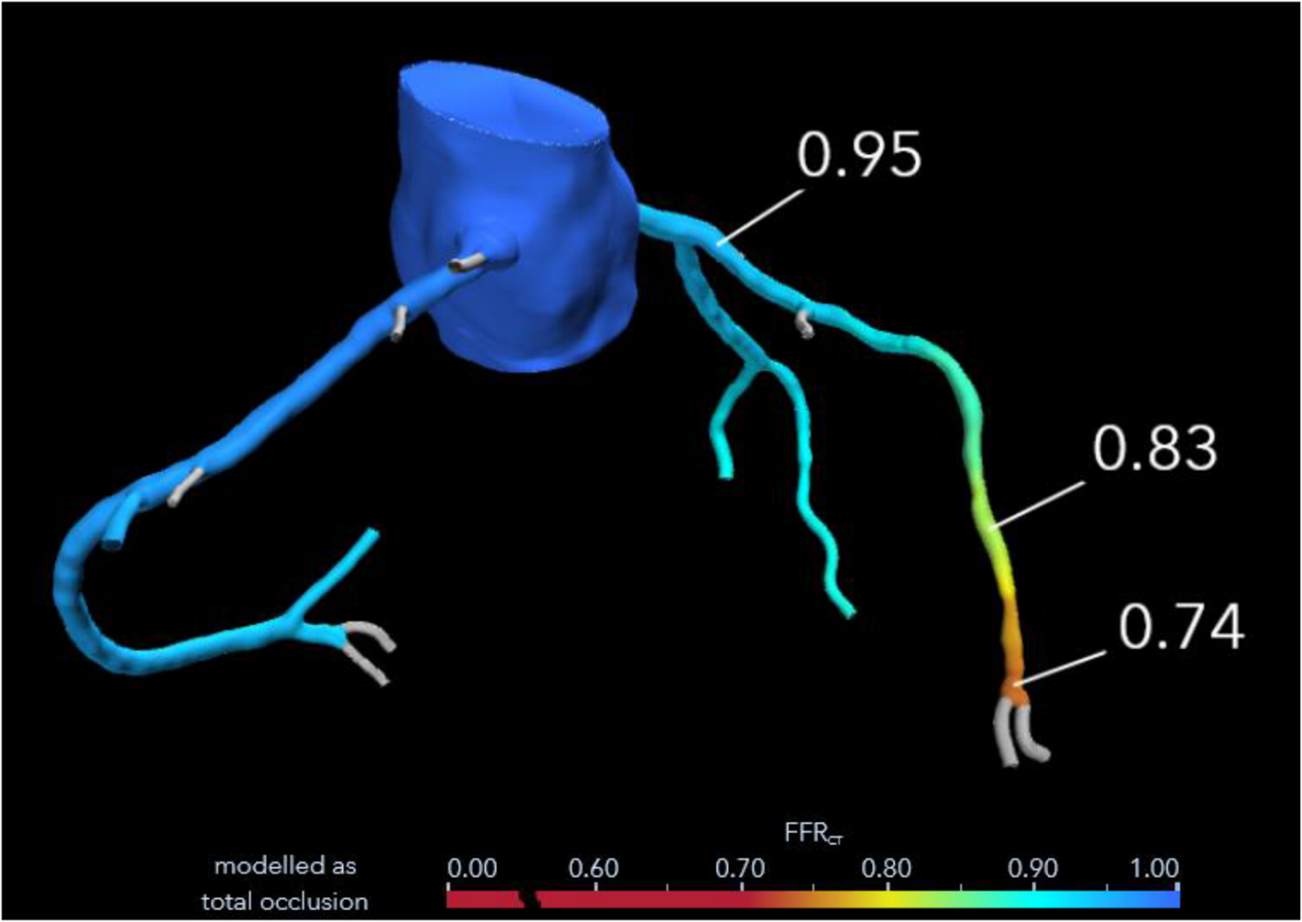
CT coronary angiogram with CT-FFR indicates distal left anterior descending coronary artery (LAD) ischemia. Right anterior oblique-cranial projection

CT pulmonary angiogram (CTPA) was performed and demonstrated no evidence of pulmonary embolism (PE); however, it showed the already known mediastinal mass. The cancer appeared to have increased in size compared with her previous CT images, and it has encased the great vessels. There was compression of the pulmonary trunk and both pulmonary arteries from a large 130 × 92 × 109 mm tumour. There was the involvement of the pleura and pericardium and, most notably, encasement and displacement of the left anterior descending artery (LAD) (Fig. 2). CT coronary angiogram (CTCA) showed displacement of the LAD with a significant reduction in the CT-FFR distal vessel indicating flow-limiting tumour compression (Figs. 3, 4, 5).

**Fig. 6:**
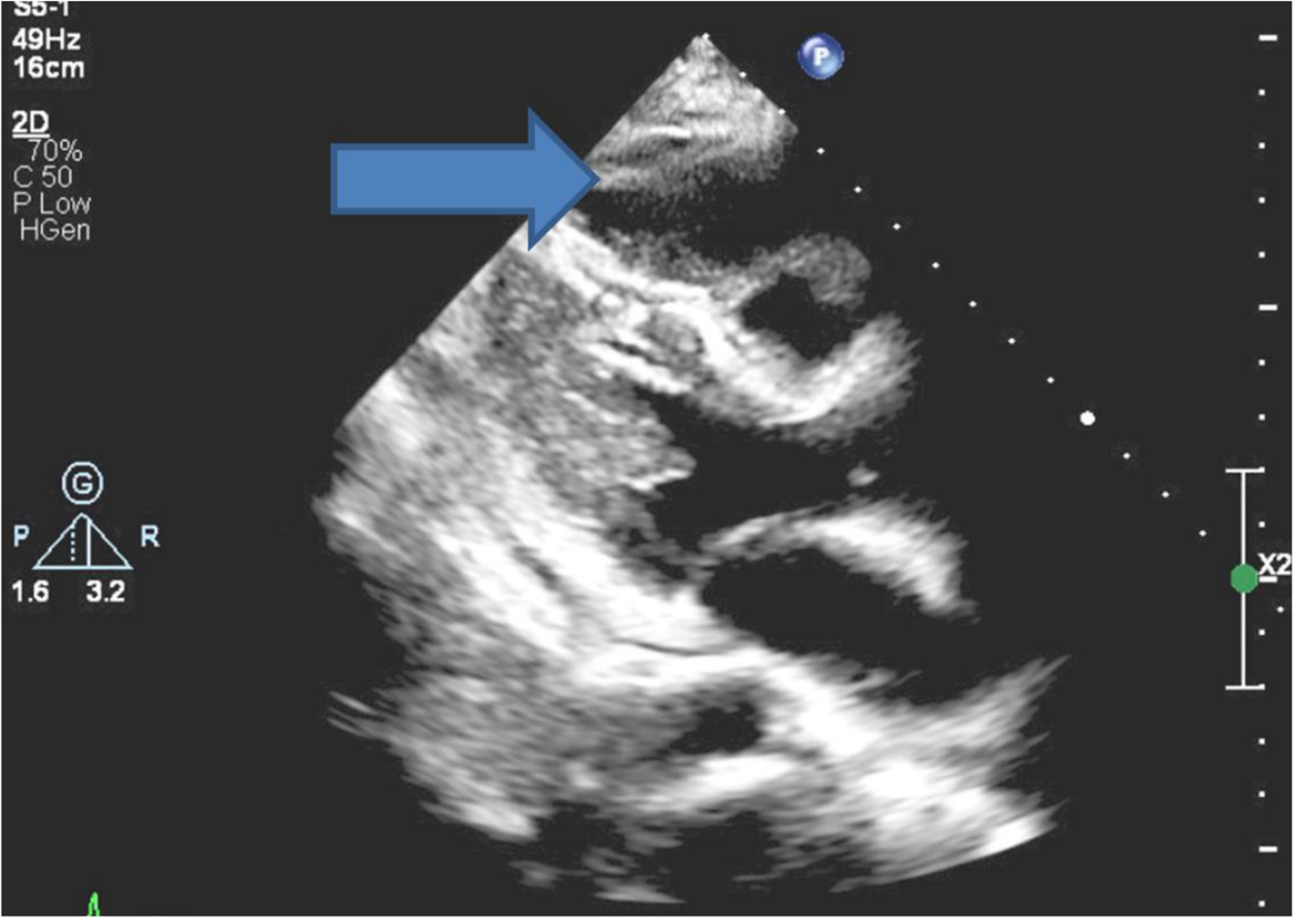
Transthoracic echocardiogram parasternal long axis view demonstrates mediastinal mass anterior to the right ventricle and compressing the right ventricular outflow tract (RVOT) (blue arrow)

She was diagnosed with acute coronary syndrome supported by serial Troponin I levels of 223 ng/L and 628 ng/L. Echocardiography showed a mass compressing the right ventricular outflow tract (RVOT) (Fig. 6) and it confirmed mild to moderate LV systolic dysfunction (45% +/− 5 LV ejection fraction by Simpson’s Biplane method) and anterolateral hypokinesia (Supplementary movie). A unifying diagnosis of LAD compression secondary to cancerous mass effect was apparent. She was commenced on dual antiplatelet therapy, glyceryl trinitrate (GTN) infusion for ongoing angina pains and subsequently transferred urgently to the local tertiary cardiac centre for further management.

Her case was reviewed and discussed at the multidisciplinary team (MDT) meeting. The consideration was for her to proceed directly to coronary angiography with a view to percutaneous coronary intervention (PCI). PCI and stenting for extrinsic coronary artery compression is an unusual undertaking with a variable success rate in the context of extrinsic tumour displacement and compression [11, 12]. However, PCI has been used to relieve coronary artery compression by other tumours with a guarded prognosis [7]; we could not find any previous report to use the PCI in the context of compressive TET. Following detailed MDT discussions between cardiologists and oncologists, the decision was made to treat this lady with palliative chemotherapy. Given the extent of the tumour invasion and failure of the initial therapy, her prognosis and outcome were poor, and the patient has been referred to the palliative care team for symptoms control.

## Conclusions

TET is a common mediastinal tumour, but cardiac involvement is rare. To the best of our knowledge, this is the first case report of a patient with TET presenting with cardiac chest pain as a consequence of encasement and displacement of the coronary artery by an enlarging TET. This case highlights the importance of cardiac and chest imaging in making the diagnosis and guiding treatment options and follow-up care.

## Supplementary Information

**Additional file 1.** Movie of transthoracic echocardiogram parasternal short axis view demonstrates regional wall motion abnormalities- hypokinesia of the anterolateral segment.

## Data Availability

Not applicable

## Abbreviations

ACS: Acute coronary syndrome
ECG: Electrocardiogram
CTCA: Computed tomography coronary angiography
CT-FFR: Computed tomography – fractional flow reserve
CTPA: Computed tomography pulmonary angiography
MDT: Multi-disciplinary team
PE: Pulmonary embolism
RVOT: Right ventricular outflow tract
TET: Thymic epithelial tumour
